# PilY1 Promotes *Legionella pneumophila* Infection of Human Lung Tissue Explants and Contributes to Bacterial Adhesion, Host Cell Invasion, and Twitching Motility

**DOI:** 10.3389/fcimb.2017.00063

**Published:** 2017-03-07

**Authors:** Julia Hoppe, Can M. Ünal, Stefanie Thiem, Louisa Grimpe, Torsten Goldmann, Nikolaus Gaßler, Matthias Richter, Olga Shevchuk, Michael Steinert

**Affiliations:** ^1^Institut für Mikrobiologie, Technische Universität BraunschweigBraunschweig, Germany; ^2^Pathology of the University Hospital of Lübeck and the Leibniz Research CenterBorstel, Germany; ^3^Airway Research Center North (ARCN), Member of the German Center for Lung ResearchBorstel, Germany; ^4^Institut für Pathologie, Klinikum BraunschweigBraunschweig, Germany; ^5^Klinikum Salzdahlumerstraße BraunschweigBraunschweig, Germany; ^6^Center for Proteomics, University of RijekaRijeka, Croatia; ^7^Helmholtz Center for Infection ResearchBraunschweig, Germany

**Keywords:** *L. pneumophila*, PilY1, human lung tissue explants, adherence, invasion, twitching motility

## Abstract

Legionnaires' disease is an acute fibrinopurulent pneumonia. During infection *Legionella pneumophila* adheres to the alveolar lining and replicates intracellularly within recruited macrophages. Here we provide a sequence and domain composition analysis of the *L. pneumophila* PilY1 protein, which has a high homology to PilY1 of *Pseudomonas aeruginosa*. PilY1 proteins of both pathogens contain a von Willebrand factor A (vWFa) and a C-terminal PilY domain. Using cellular fractionation, we assigned the *L. pneumophila* PilY1 as an outer membrane protein that is only expressed during the transmissive stationary growth phase. PilY1 contributes to infection of human lung tissue explants (HLTEs). A detailed analysis using THP-1 macrophages and A549 lung epithelial cells revealed that this contribution is due to multiple effects depending on host cell type. Deletion of PilY1 resulted in a lower replication rate in THP-1 macrophages but not in A549 cells. Further on, adhesion to THP-1 macrophages and A549 epithelial cells was decreased. Additionally, the invasion into non-phagocytic A549 epithelial cells was drastically reduced when PilY1 was absent. Complementation variants of a PilY1-negative mutant revealed that the C-terminal PilY domain is essential for restoring the wild type phenotype in adhesion, while the putatively mechanosensitive vWFa domain facilitates invasion into non-phagocytic cells. Since PilY1 also promotes twitching motility of *L. pneumophila*, we discuss the putative contribution of this newly described virulence factor for bacterial dissemination within infected lung tissue.

## Introduction

*Legionella pneumophila* is the causative agent of the Legionnaires' disease, a severe form of pneumonia (Fraser et al., 1977; McDade et al., 1977; Fields et al., 2002). Upon transmission to the respiratory tract through aerosols containing *Legionella*, the bacteria enter and replicate within alveolar macrophages and epithelial cells (Horwitz and Silverstein, 1980; Mody et al., 1993; Jäger et al., 2014). The cellular internalization of *L. pneumophila* can be enhanced by the presence of antibodies and complement. The major outer membrane protein (MOMP) of *L. pneumophila* binds complement component C3 and C3i and mediates the uptake of the bacteria via the complement receptors CR1 and CR3 of macrophages. Phagocytosed *L. pneumophila* recruit compartments from the endoplasmatic reticulum (ER), modulate the host phosphoinositide metabolism, modify the host endocytic pathway, intercept vesicle trafficking and avoid fusion with lysosomes (Shevchuk et al., 2014). The development of the *Legionella*-containing vacuole (LCV) is pre-dominantly orchestrated by the Dot/Icm type IV secretion system, which is known to export numerous bacterial effector proteins into the host cell cytoplasm (Horwitz, 1983; Kagan and Roy, 2002; Isberg et al., 2009; Hubber and Roy, 2010). Bacterial attachment and entry into host cells are considered to be a pre-requisite for this pathogen-directed host cell modulation (Roy et al., 1998; Wiater et al., 1998; Hilbi et al., 2001; Charpentier et al., 2009). Several determinants of *L. pneumophila* are known to contribute to adherence and entry into different host cell types, including type IV pili, Hsp60, the structural toxin RtxA, the intergrin analog LaiA and the GAG binding protein Lcl and the adenylate cyclase LadC (Garduño et al., 1998; Stone and Abu Kwaik, 1998; Cirillo et al., 2001; Chang et al., 2005; Newton et al., 2008; Duncan et al., 2011).

In a previous study, we screened a *L. pneumophila* mini-Tn10 transposon library for mutants that fail to avoid fusion of their respective LCV with lysosomes (Shevchuk et al., 2014). The range of the identified mutants indicated that interference with lysosomal degradation is multifactorial. Several mutants with different insertions in the Lpc2666 gene exhibited significantly higher co-localization with lysosomal compartments and reduced replication rates in macrophages and protozoa (Shevchuk et al., 2014). The sequence analysis revealed that Lpc2666 encodes for a type IV fimbrial biogenesis PilY1-like protein that shares homology with the C-terminal domain of PilY1 of *Pseudomonas aeruginosa* and the PilC1/2 of *Neisseria gonorrhoeae, Neisseria meningitides*, and *Kingella kingae*. The PilC proteins of *K. kingae* and *Neisseria* species have already been characterized as type IV pili biogenesis factors and are known to be involved in adherence to epithelial cells (Rudel et al., 1995a,b; Scheuerpflug et al., 1999; Porsch et al., 2013). The PilY1 of *P. aeruginosa* has also been shown to be essential for type IV pilus assembly and evidently contributes to cell adhesion and virulence (Bohn et al., 2009; Heiniger et al., 2010). In addition, it has been demonstrated that the PilY- or PilC-like proteins are required for pilus stability. Accordingly, mutations in the respective genes result in the loss of the type IV pilus dependent twitching motility. Moreover, *P. aeruginosa* PilY1 participates in the regulation of a type IV pilus independent motility (Wolfgang et al., 1998; Morand et al., 2004; Bohn et al., 2009; Kuchma et al., 2010; Porsch et al., 2013).

In the present study, we analyzed the sequence and domain composition of the *L. pneumophila* PilY1. We ascertained PilY1 as an outer membrane protein that is expressed during the stationary growth phase of the bacteria. Since the *L. pneumophila* PilY1 knockout mutant exhibited defects in twitching motility as well as in host cell adherence, invasion and intracellular replication, we hypothesize that PilY1 mediates extra- and intracellular virulence mechanisms which are required for the efficient infection of human lung tissue explants (HLTEs).

## Materials and methods

### Cultivation of bacteria and eukaryotic cells

*L. pneumophila* Corby strains and mutants were routinely cultured on buffered charcoal-yeast extract (BCYE) agar for 3–5 days. Liquid cultures were inoculated in buffered yeast extract (YEB) medium and grown at 37°C with agitation at 180 rpm to an OD600 of 3.0 with 12.5 μg/ml chloramphenicol and 500 μM IPTG or 20 μg/ml kanamycin if required. Human alveolar epithelial A549 cells (DSMZ, ACC-107) were grown in DMEM and the human monocyte cell line THP-1 (DSMZ, ACC-16) in RPMI 1640 medium, both supplemented with 10% FCS and 4.5 mM glutamine at 37°C and 5% CO_2_. For the experiments, the A549 cells were seeded 18 h before infection into 24-well tissue culture plates (GreinerBioOne) at a density of 5 × 10^5^ cells/well. The THP-1 cells were seeded in 96-well tissue culture plates (TTP) at a density of 10^5^ cells/well and differentiated with phorbol myristate acetate (PMA; Sigma) to a final concentration of 100 nM for 48 h.

### Site-directed mutagenesis of *pilY1* and construction of complementation strains

The *L. pneumophila* Corby *pilY1* knockout mutant was generated by allelic exchange of the chromosomal *pilY1* gene for a kanamycin resistance cassette. For this purpose, a PCR fragment carrying the antibiotic resistance gene flanked by a 1 kb homologous region to the target locus was generated by a three-step amplification procedure (Derbise et al., 2003). In the first step, the flanking upstream and downstream regions of the *pilY1* gene and the kanamycin cassette were amplified independently. The primers PilYupR and PilYdownF contain an extension of 20 nucleotides homologous to the kanamycin resistance cassette. In the second step, the resulting three PCR products were mixed at equimolar concentrations and subjected to a second PCR to generate the *pilY1* knockout construct, which was ligated into the pGEM®-T Easy vector. The third step was required to obtain large amounts of the desired DNA. The DNA was introduced into *L. pneumophila* by natural transformation as described previously with modifications (Sexton and Vogel, 2004; Schunder et al., 2010). Briefly, 1 ml of an exponentially grown overnight culture was incubated with 1 μg of the PCR product for 3 days at 30°C without agitation. Subsequently, bacteria were grown on antibiotic selective media for 4 more days at 37°C with 5% CO_2_. Screening for mutants obtained by homologous recombination was performed by PCR using the primer pairs PilYconfF/R. For complementation of the *pilY1* mutant, the full length *pilY1* PilY and vWFa domains were amplified using the primers pilYcomF/R, PilYvWFF/comR, and PilY-vWFF/comR/domR. The resulting PCR fragments were digested with *XbaI* and *EcoRI* and cloned into the pXDC/pMMB207C vector at the position of the *blaM* orf (Charpentier et al., 2009) and electroporated into the strain *L. pneumophila* Corby Δ*pilY1*. For a complete list of plasmids and primers prefer to Table 1. Complementation of the strains on protein level, and the correct localization on the surface of the bacteria were confirmed by western blot using polyclonal rabbit α-PilY1 antibodies directed against a central peptide of 12 amino acids (Supplementary Information and Supplementary Figures 1 and 4).

**Table 1:** **Plasmids and Primers used in this study**.

**Plasmid**	**Description**	**References**
pXDC WTPilY	pXDC61-derived vector expressing PilY1	This study
pXDC ΔPilYdom	pXDC61-derived vector expressing PilY1 lacking the PilY domain (Δaa 600–1169); Cm^*r*^	This study
pXDC ΔvWFa	pXDC61-derived vector expressing PilY1 lacking the vWFa domain (Δaa 405–526); Cm^*r*^	This study
pXDC61	pMMB207C-derived vector; Cm^*r*^	Charpentier et al., 2009
pGEM-T	Shuttle vector	Promega
pGEM-T PilYKm	Vector for knockout of pilY gene; Km^*r*^	This study
**Primer**	**5′–3′Sequence**	**References**
PilYup F	GTTGAATATGGCTCATAGCGTCCATGATAATCAAAACC	This study
PilYup R	CTCAAACCCAACTTTACAAAGC	This study
PilYdown F	TAAAAACCTTGCAGGAATACGG	This study
PilYdown R	TTGTAACACTGGCAGAGGCAAATTCAATAGAGGATACCC	This study
Km F	TCTGCCAGTGTTACAACCAATT	This study
Km R	ATGAGCCATATTCAACGGGA	This study
PilYconf F	GGCAGATTAATTGTAATGTCAGTGT	This study
PilYconf R	CCAGGATTTTCATTAGTCGAGTTAAT	This study
PilYcom F *XbaI*	GCGCGTCTAGATTGAATTTGCCACCAGCC	This study
PilYcom	GCGCGGAATTCGTTTTGATTATCATGGACGC	This study
R *EcoRI*	GCGCGGAATTCTATCATGGACGCTTTAAC	This study
PilYvWF F *XbaI*	TGGTCAACTAACCGAAAG	This study
PilYdom R		
PilY-vWF R	CGGTTAGTTGACCATTGCGGACAATTGCCACT	This study
SP6	TATTTAGGTGACACTATAG	Promega
T7	TAATACGACTCACTATAGGG	Promega
pXDC F	TTGACAATTAATCATCGGC	This study
pXDC R	CTGTATCAGGCTGAAAATC	This study

### Cellular localization of PilY1 and its variants using triton X-100 solubility

Cell fractionation was performed as previously described with modifications (Vincent et al., 2006; Kuchma et al., 2007, 2010). Cells were resuspended in 50 mM Tris-HCl [pH 8] with lysozyme (0.2 mg/ml^−1^) and DNAse (1 μg/ml) and lysed in a French pressure cell (14,000 PSI). Extracts were then centrifuged 10 min at 10,000 g to remove unlysed cells and obtain whole-cell lysates. To separate the soluble cytoplasmic fraction from the total membrane (TM) fraction, whole-cell lysates were centrifuged at 100,000 g for 1 h at 4°C. The membrane pellet was resuspended in 50 mM Tris-HCl [pH 8] to yield the TM fraction. The inner membranes were solubilized by the addition of 20 mM MgSO_4_ and 1% Triton X-100; the outer membranes were collected by additional centrifugation at 100.000 g for 1 h. To yield the outer-membrane fraction, pellets were resuspended in 50 mM Tris-HCl [pH 8] and SDS loading buffer. Protein concentrations of each fraction were determined using a Roti®-Nanoquant protein quantification assay (Roth) according to the manufacturer's instructions. For each cellular fraction, 25 μg of total protein were immunoblotted with antibodies which recognize the PilY protein, and detected by using a horseradish conjugated secondary antibody (Dianova) and the Amersham ECL Western Blotting Detection Reagent. The rabbit polyclonal PilY antibody was generated against a peptide corresponding to the PilY 359–370 aa (Eurogentec). The integrity of the cellular fractions was confirmed using the antibodies which recognize the inner membrane type I signal peptidase LepB and the outer membrane protein MOMP (Vincent and Vogel, 2006). Alkaline phosphatase-conjugated antibodies (Thermo Scientific) were used for detection.

### PilY1 expression analysis

To determine PilY expression, the *L. pneumophila* wild type strain was grown in YEB broth at 37°C with agitation at 180 rpm. PilY1 expression was tested with 10^9^ bacteria respectively from the exponential, early stationary, stationary and late stationary growth phases. The bacteria were lysed with 7 M Urea, 20 mM Tris-HCl [pH 9], 100 mM DTT and 1% Triton X-100. Equal cell amounts were immunoblotted with antibodies that recognize the PilY protein and detected as described above. Western Blot analysis was performed using the antibody for FlaA as a marker for the stationary growth phase expression, and Mip as control for equal cell amounts (Helbig et al., 1995; Heuner et al., 1995). Alkaline phosphatase conjugated antibody (Thermo Scientific) was used for detection.

### Infection of human lung tissue explants

For infection, tumor-free pulmonary tissue samples, which had been obtained from surgery patients, were infected with 10^7^ bacteria/ml of early stationary phase strains of *L. pneumophila*. The tissue samples were incubated in RPMI 1640 with 10% FCS, 20 mM HEPES, and 1 mM sodium pyruvate at 37°C and 5% CO_2_ for up to 48 h as described previously (Jäger et al., 2014). For the determination of the CFU per g tissue, samples from six donors were infected. At the indicated time points after infection, samples were weighed, homogenized and dilutions were plated on BCYE agar and incubated at 37°C with 5% CO_2_ for 4 days.

### Infection assays with THP-1 macrophages and A549 epithelial cells

The human monocytic cell line THP-1 and the human alveolar epithelial cell line A549 were pre-treated as described above and infected with *L. pneumophila* strains from the early stationary phase with a MOI 1 (multiplicity of infection) for THP-1 cells and a MOI 100 for A549 cells for 2 h in 5% CO_2_ at 37°C. Cells were then washed with PBS to remove extracellular bacteria. The A549 cells were additionally treated with 100 μg/ml gentamicin for 1 h. At indicated time points following infection, cells were lysed with 0.01% Triton X-100, serial dilutions were plated on BCYE agar and incubated at 37°C with 5% CO_2_ for 4 days in order to determine the CFU/ml.

### Adhesion and invasion assay

The experiments were performed with confluent A549 cells and differentiated THP-1 macrophages as described above. Stationary phase *L. pneumophila* strains were added to THP-1 macrophages (MOI 20) and A549 cells (MOI 100). The infections were synchronized by centrifugation at 300 g for 5 min. For the adhesion assay, the cells were pre-incubated with 10 μM cytochalasin B for 1 h to prevent phagocytosis. After 30 min of co-incubation with THP-1 macrophages and after 1 h co-incubation with A549 cells, the monolayers were washed twice with PBS to remove non-adherent bacteria and lysed with 0.01% Triton X-100. Serial dilutions of the inoculum and of bacteria recovered from lysed cells were plated on BCYE agar. Results are expressed as the ratio of adherent bacteria compared to the inoculum. For the invasion assay a differential staining protocol was performed. For this, the cells were infected for 1 h (THP-1) or 2 h (A549) with bacteria that were labeled with rhodamine. Unbound bacteria were removed by two washes of PBS, and the cells were fixed with 4% PFA over night at 4°C. Following fixation, PFA was removed by three washes with PBS containing 50 mM glycin, and the samples were blocked using 10% NHS in SorC-buffer. The extracellular bacteria were additionally stained using a polyclonal rabbit α-*L. pneumophila* antibody (ABIN23774) followed by an Alexa Fluor® 488-coupled goat α-rabbit antibody (Abcam, ab15007). Mounted samples were analyzed using a Leica SP8 confocal laser scanning microscope. For each strain, at least 50 cells with associated bacteria were analyzed in three independent experiments, and the ratio of internalized bacteria were calculated. Rhodamine labeling of bacteria was performed as described previously (Shevchuk et al., 2014).

### Motility assays

Surface motility was monitored as previously described (Coil and Anné, 2009; Stewart et al., 2009). Briefly, 10 μl of early stationary phase cultures of *Legionella* strains were dropped onto fresh BCYE plates containing 1.6% (twitching motility) or 0.5% (sliding motility) agar. The inoculated plates were incubated at 30°C and growth was observed for the next 14 days. Plates were photographed, and the area in which migration had occurred was calculated with Adobe Photoshop 7.0.1.

### Statistical analysis

All experiments were performed in duplicate and repeated at least three times. Statistical analysis was performed using Student's *t*-test in GraphPad Prism 5.0. Differences were considered significant at a *p* ≤ 0.05.

## Results

### Sequence and domain composition analyses of PilY1

**Figure 1:**
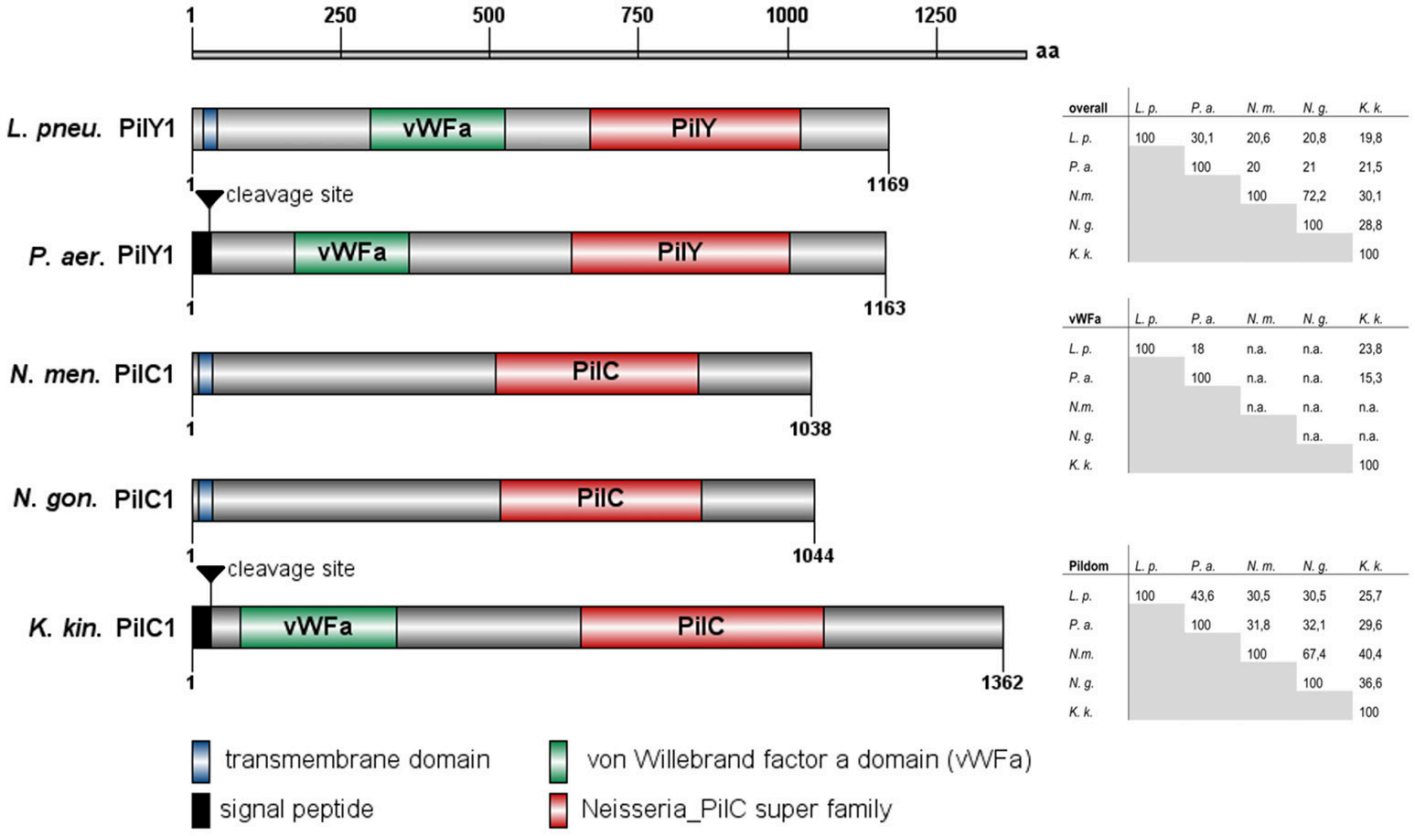
**Predicted domain organization of *L. pneumophila* Corby PilY1 protein and that of homologous proteins from *P. aeruginosa PAK, Neisseria meningitidis, Neisseria gonorrhoeae*, and *Kingella kingae*.** Sequence analysis and diagrams were performed with SMART (Simple Modular Architecture Research Tool) and IBS (Illustrator of Biological Sequences; Letunic et al., 2015; Liu et al., 2015). The degree of homology between the single proteins is also shown in form of matrices for the whole protein, and the single domains for each protein (n.a., not applicable).

Given the importance of PilY and PilC proteins in type IV pilus biogenesis and stability, including pilus extension and retraction in several bacterial pathogens (Orans et al., 2010; Johnson et al., 2011; Cheng et al., 2013; Porsch et al., 2013), we performed a sequence-based domain annotation of the *L. pneumophila* PilY1 protein. Based on sequence homology with *P. aeruginosa* PilY1, *N. gonorrhoeae, N. meningitides*, and *K. kingae* PilC, we predicted the PilY1 structure of *L. pneumophila* as depicted in Figure 1. At the N-terminus the PilY1 protein contains a transmembrane domain with a putative signal peptide cleavage site, similar to the Sec secretion signal peptide of *P. aeruginosa* PilY1 (Lewenza et al., 2005; Kuchma et al., 2010). Moreover, a 200 amino acid N-terminal region with homology to the von Willebrand factor A (vWFa) domain was identified, which is also present in the PilY1 of *P. aeruginosa* and the PilC1 of *K. kingae*. The highest sequence similarity within this protein class was found in the C-terminal PilY or PilC domain. With 36% aa identity and 48% aa similarity, the *L. pneumophila* PilY domain is most closely related to the PilY domain of *P. aeruginosa*.

### PilY1 is expressed during stationary growth phase

**Figure 2:**
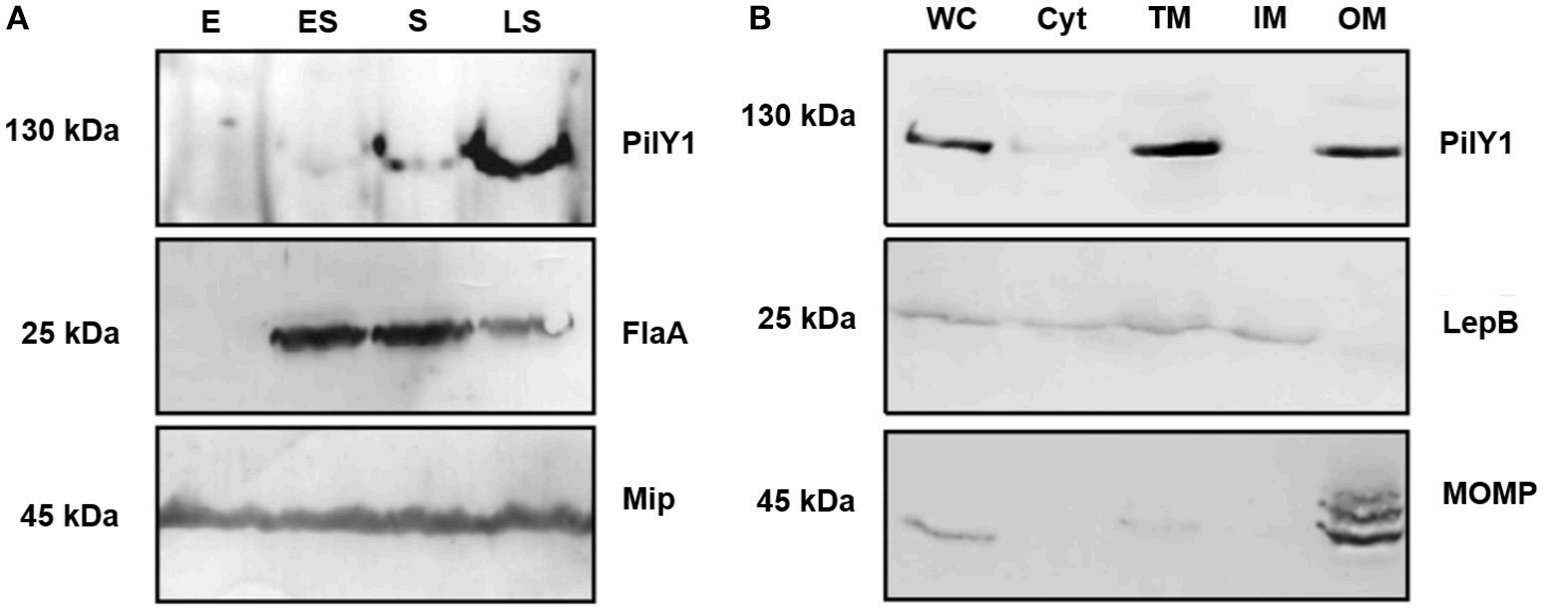
**Growth phase dependent expression of *L. pneumophila* Corby PilY1 and its subcellular localization. (A)** Immunoblotting of bacterial whole-cell lysates from exponential (E), early stationary (ES), stationary (S), and late stationary (LS) growth phases with antibodies specific for PilY1, Mip (constitutive expression) and FlaA (expression during stationary growth phase). PilY1 expression was only detected in the stationary growth phase of *L. pneumophila* which represents the transmissive phase of infection. **(B)** Immunoblotting of subcellular fractions of *L. pneumophila* Corby from the stationary growth phase. Whole cell lysate (WC), cytoplasmic (Cyt), total membrane (TM), inner membrane (IM), and outer membrane (OM) fractions were immunoblotted with antibodies against PilY1, LepB (inner membrane signal peptidase), and MOMP (major outer membrane protein).

*L. pneumophila* employs a biphasic life cycle, where it alternates between an infectious, non-replicating form, which promotes transmission to and manipulation of new host cells, and an intracellular, replicative phase, which downregulates specific virulence traits. The expression of virulence genes correlate with the post-exponential and stationary growth phases of the bacteria and can be modeled in liquid culture medium (Molofsky and Swanson, 2004). Transcriptional profiling revealed that *pilY1* expression is induced during the transmissive, post-exponential growth phase (Brüggemann et al., 2006; Faucher et al., 2011). In the present study, we compared the PilY1 expression of whole-cell lysates of *L. pneumophila* Corby during exponential (E), early stationary (ES), stationary (S), and late stationary (LS) growth phase by Western blot analysis (Figure 2A). The discrimination between exponential and stationary growth phases of *L. pneumophila* was confirmed using a flagellin (FlaA) specific antibody, since flagellation is a prominent feature of the stationary growth phases. As a control for equal cellular amounts in the lysates, the constitutively expressed macrophage infectivity potentiator (Mip) was used for immunoblotting. As shown in Figure 2A, PilY1 expression was up-regulated during stationary growth which represents the transmissive phase of infection.

### PilY1 localizes in the outer membrane fraction

According to the signal peptide prediction program SignalP, the PilY1 protein harbors an N-terminal signal peptide and the subcellular localization prediction tool PSORTB 3.0.2 predicts that the PilY1 protein localizes in the outer membrane (Emanuelsson et al., 2007; Yu et al., 2010). To determine the cellular localization of the PilY1 protein experimentally, *L. pneumophila* cells from the stationary growth phase were fractionated utilizing Triton X-100 solubility and ultracentrifugation. Most Gram-negative bacterial inner membrane proteins are soluble in the non-ionic detergent Triton X-100, whereas outer-membrane proteins are typically insoluble (Nikaido, 1994; Vincent et al., 2006). With this approach whole cell lysates (WC) of *L. pneumophila* were separated into cytoplasmic (Cyt), total membrane (TM), inner membrane (IM), and outer membrane (OM) fractions. Western blotting revealed the presence of PilY1 in the whole cell lysate (WC), total membrane (TM), and outer membrane (OM) fractions (Figure 2B). The integrity of the membrane fractions was confirmed by detection of the inner membrane signal peptidase LepB and the major outer membrane protein MOMP (Bellinger-Kawahara and Horwitz, 1990; Chen et al., 2007). In summary, these results suggest that PilY1 is located in the outer membrane fraction of *L. pneumophila*.

### *L. pneumophila* PilY1 promotes infection of human lung tissue explants

**Figure 3:**
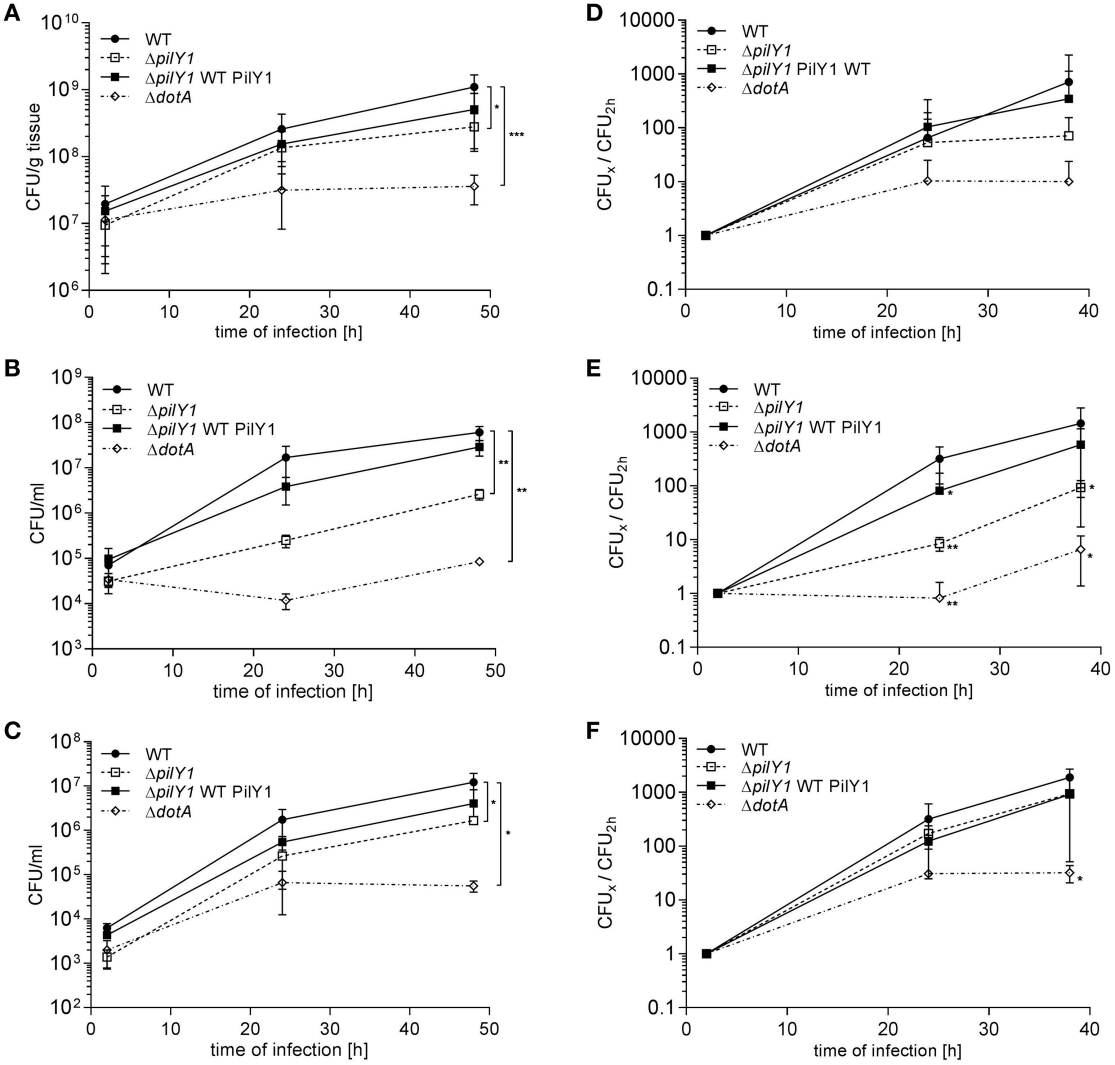
**Effect of PilY1 on *L. pneumophila* Corby infection of HLTEs, THP-1 macrophage-like cells and A549 lung epithelial cells. (A,D)** HLTEs, **(B,E)** THP-1 macrophages, and **(C,F)** A549 cells were infected with *L. pneumophila* Corby wild type strain (WT), the *pilY1-*negative mutant (Δ*pilY1*), the *pilY1* complemented mutant (Δ*pilY1* WTPilY1), and the DotA deficient mutant strain (Δ*dotA*). CFUs were determined by plating serial dilutions on BCYE agar plates at the indicated time points. The replication rates during the same infection set-ups were calculated and plotted accordingly for HLTEs **(D)** THP-1 macrophages **(E)** and A549 cells **(F)** by dividing the CFU of the respective time point (CFU_x_) by the CFU of the 2 h time point (CFU_2h_). The graphs show means and standard deviations from duplicate infections with tissues from six donors **(A)** and from three independent cellular infections in duplicate **(B,C)**. Significance was assessed by applying an unpaired two-sided Student's *t*-test (^*^p ≤ 0.05, ^**^p ≤ 0.01, ^***^p ≤ 0.001).

Human lung tissue explants (HLTE) with their multitude of cell types and extracellular components are well-suited for a comprehensive investigation of extra- and intracellular pathogenicity mechanisms of *L. pneumophila* at a unique level of complexity (Jäger et al., 2014). Since PilY1 is expressed during the transmissive phase of *L. pneumophila*, we analyzed the contribition of this factor that is associated with the outer membrane during HLTE infection. Tumor-free pulmonary tissue samples had been obtained from surgery patients and inoculated with the *L. pneumophila* Corby wild type strain, the PilY1-negative *L. pneumophila* Δ*pilY1* mutant, the PilY1 domain-positive *L. pneumophila* Δ*pilY1* WTPilY1 complemented mutant, and the DotA-negative *L. pneumophila* Δ*dotA* mutant. The CFUs/g tissue were determined during 48 h postinoculation (Figure 3A). The *L. pneumophila* Δ*pilY1* mutant showed a significant growth defect in lung tissue with a four-fold reduced bacterial load compared to the *L. pneumophila* wild type strain 48 h post infection. Complementation restored the ability of the mutant to replicate within the infected tissue to a large extent. The number of DotA-negative bacteria, which cannot replicate within host cells, did not increase significantly. Interestingly, when the replication rates of the strains were compared by dividing the CFUs of respective time points (CFU_x_) by the initial CFU at 2 h post infection (CFU_2h_), no significant differences could be observed (Figure 3D). In summary, these results demonstrate that PilY1 promotes the infection of human lung tissue in an early state of establishing the infection.

### PilY1 affects intracellular replication of *L. pneumophila*

Intracellular replication in macrophages and epithelial cells is essential for *L. pneumophila* infection of lung tissue (Mody et al., 1993; Gao et al., 1998; Jäger et al., 2014). In a previous screening of mini-Tn10 transposon mutants we showed that the PilY1-negative mutants (Lpc2666) were attenuated in their ability to replicate intracellularly within U937 macrophage-like cells (Shevchuk et al., 2014). To assess the impact of *pilY1* on intracellular replication during the infection of HLTEs more specifically, THP-1 macrophage-like cells and A549 lung epithelial cells were infected with the *L. pneumophila* WT, the Δ*pilY1* mutant and the Δ*pilY1* mutant complemented with PilY1. In both host cell types, the overall intracellular replication of the Δ*pilY1* mutant was significantly attenuated after 24 and 48 h compared with the *L. pneumophila* wild type strain (Figures 3B,C). With a 40-fold reduction after 48 h, the strongest intracelluar growth defect was observed in the THP-1 macrophage-like cells. A closer look at the replication rates in both cell types revealed that the pronounced attenuation in replication in THP-1 cells was indeed due to a defect in intracellular multiplication (Figure 3E). In A549 cells the reduced infection was due to a lower intracellular load with bacteria rather than to a change in the replication rate (Figure 3F). Either growth defect was repaired by the complementation with the full length *pilY1* gene. These results show that PilY1 contributes to the intracellular growth of *L. pneumophila* in macrophages and epithelial cells by different means. This cell type dependent effect regarding intracellular replication could also be confirmed by immunofluorescence microscopy. In THP-1 macrophages the Δ*pilY1* mutant co-localized with the phagolysosomal marker LAMP-1 to a significantly greater extent than wild type bacteria in. On the contrary, no difference in LAMP-1 co-localization was observed in A549 cells (Supplementary Figure 3).

### PilY1 contributes to host cell adherence and invasion

**Figure 4:**
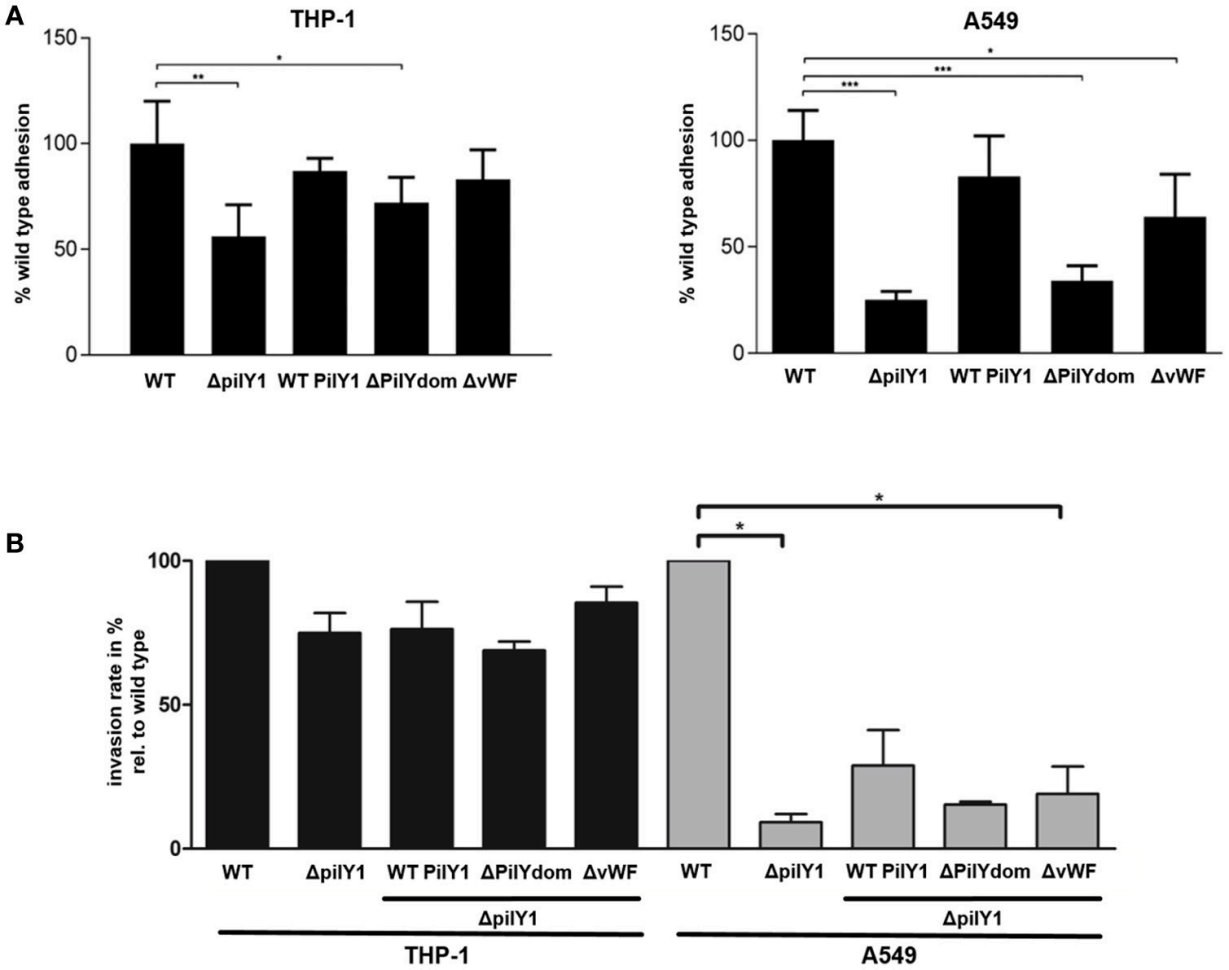
**Contribution of PilY1 domains to host cell adherence and invasion. (A)** Adherence to THP-1 macrophage like cells and A549 epithelial cells was analyzed with the *L. pneumophila* Corby wild type strain (WT), the *pilY1-negative* mutant (Δ*pilY1*), the complemented *pilY1* mutants expressing the wild type PilY1 (Δ*pilY1* WTPilY1), the PilY1 lacking the C-terminal PilY1 domain (ΔPilYdom) and the PilY1 lacking the vWFa domain (ΔvWFa). Bacterial adhesion was determined 30 min (THP-1) or 1 h (A549) after inoculation and is presented as a ratio of adhering bacteria to the total number of inoculated bacteria. Bacterial uptake by phagocytosis was blocked with Cytochalasin B. **(B)** Invasion of host cells was determined 1 h (THP-1) or 2 h (A549) after inoculation with rhodamine labeled bacteria. Extracellular bacteria additionally were stained with polyclonal α-*L. penumophila* antibodies and detected with an Alexa Fluor® 488-coupled secondary antibody. Presented is the ratio of intracellular bacteria to the total number of cell-associated bacteria. Data represent the means and standard deviations from duplicates of three independent experiments (*t*-test, ^*^p ≤ 0.05, ^**^p ≤ 0.01, ^***^p ≤ 0.001).

Attachment to and invasion of host cells are critical steps in the cellular infection cycle of *L. pneumophila* and a wide variety of factors which are important for these processes have been identified, including type IV pili (Stone and Abu Kwaik, 1998; Cianciotto, 2001; Molmeret et al., 2004). To further dissect the effect on intracellular growth, we examined the specific contribution of *pilY1* to adhesion and invasion of THP-1 macrophage-like and A549 lung epithelial cells. For the determination of adherent *L. pneumophila*, phagocytosis was prevented with the actin polymerization inhibitor Cytochalasin B. The adhesion assay revealed that the adherence of the Δ*pilY1* mutant to THP-1 macrophage-like and A549 lung epithelial cells is decreased by ~50% compared to the *L. pneumophila* wild type phenotype (Figure 4A). Complementation in *trans* of the Δ*pilY1* mutant with the full length *pilY1* gene restored adherence, while the truncated form of PilY1, lacking the C-terminal PilY domain (ΔPilYdom), was not able to restore the adherence defect. The complementation with a deletion variant of PilY1, lacking the vWFa domain (ΔvWFa), revealed that this domain is dispensable for adhesion of *L. pneumophila* to THP-1 macrophage-like cells.

In order to evaluate whether invasion of the bacteria was also affected by the deletion of *pilY1* differential immunofluorescence staining was performed, and the ratio of intracellular bacteria to total cell-associated bacteria was determined. Here, again cell type dependent effects could be observed, since invasion of A549 lung epithelial cells was significantly affected and reduced by 90%. In case of THP-1 macrophage-like cells no significant reduction was measured. In case of A549 cells, complementation with the vWFa domain improved the invasion efficiency (Figure 4B).

### PilY1 influences surface motility of *L. pneumophila*

**Figure 5:**
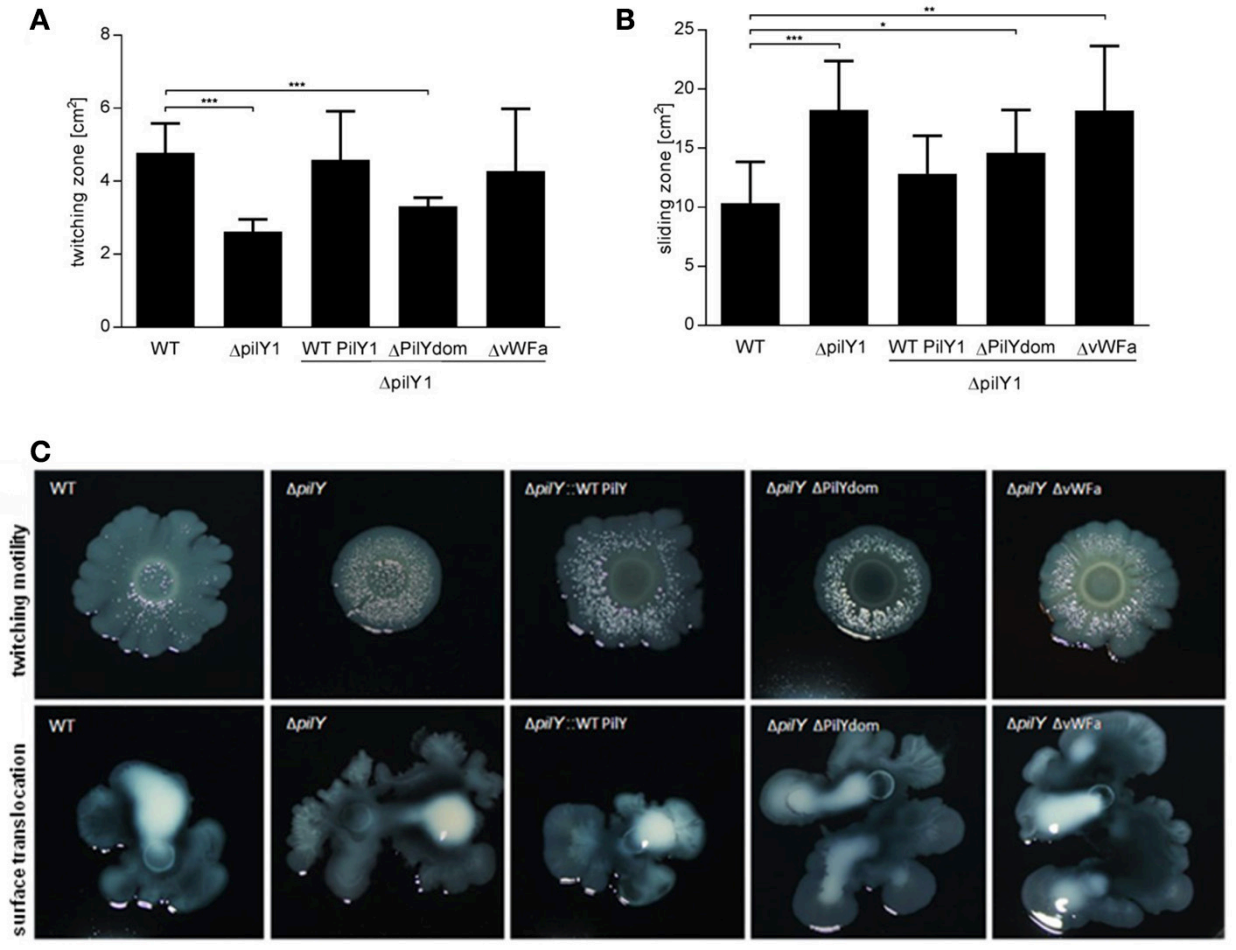
**Contribution of PilY1 domains to twitching and sliding motility**. For **(A)** twitching and **(B)** sliding motility assays stationary phase liquid cultures of *L. pneumophila* Corby wild type (WT), the *pilY1-negative* mutant (Δ*pilY1*), the complemented *pilY1* mutant strains expressing the wild type PilY1 (Δ*pilY1* WTPilY1), the PilY1 lacking the C-terminal PilY1 domain (ΔPilYdom) and the PilY1 lacking vWFa domain (ΔvWFa) were spotted on BCYE agar plates and grown at 30°C for 14 days. **(C)** Images of twitching motility and sliding translocation are representatives of three independent experiments. The central raised ring represents the initial inoculum and the outer rings show the motility zone. Migration areas were calculated with Adobe Photoshop 7.0.1. (*t*-test ^*^p ≤ 0.05, ^**^p ≤ 0.01, ^***^p ≤ 0.001).

In addition to adhesion, invasion, and intracellular replication, lung infection depends on dissemination of the pathogen within the tissue. With respect to *L. pneumophila* twitching motility, a type IV pilus-mediated movement and sliding motility, a flagellum- and pilus-independent translocation, which is facilitated by secreted surfactant, were described (Coil and Anné, 2009; Stewart et al., 2009). Analogous to twitching and sliding motility assays, which were previously performed, we examined the contribution of PilY1 to both motility processes (Figures 5A–C). The motility of *L. pneumophila* was quantified by measuring the peripheral motility zones of bacterial colonies grown on agar plates. Interestingly, the Δ*pilY1* mutant exhibited a significantly reduced twitching motility compared to the wild type strain (Figure 5A). In complementation assays, the strains expressing PilY1 (WT PilY) or a truncated form with a deletion of the vWFa domain (ΔvWFA) revealed the wild type phenotype. However, the *L. pneumophila* mutant expressing a PilY1 protein without the C-terminal PilY domain (ΔPilYdom) was unable to complement the twitching motility defect of the Δ*pilY1* mutant. In contrast to the colony morphology of the wild type strain, the border in the motility zone of the Δ*pilY1* mutant was very smooth (Figure 5C). This different colony morphology was also observed for the complemented strain lacking the C-terminal PilY domain (ΔPilYdom).

In the sliding motility assay, the spreading area of the Δ*pilY1* mutant was greater than that of the wild type strain (Figures 5B,C). In addition to an enhanced number and size of the lobes in the sliding motility zone, we also observed increased surfactant secretion on the agar surface. The genetic complementation demonstrated that only the mutant strains expressing PilY1 exhibited the smaller wild type motility. Complementation with the ΔPilYdom protein resulted in an intermediate phenotype, while complementation without the vWFa domain (ΔvWFa) resulted in sliding motility similar to that of the Δ*pilY1* mutant phenotype. In conclusion, these data indicate that PilY1 positively influences twitching motility, but confines sliding motility.

## Discussion

PilY1 proteins are conserved within many genera of gamma- and beta-proteobacteria, including pathogens with a broad host spectrum (Rahman et al., 1997; Morand et al., 2001, 2004; Kehl-Fie et al., 2008; Siryaporn et al., 2014). Sequence and domain composition analyses revealed that the PilY1-like protein of *L. pneumophila* has the highest homology to the *P. aeruginosa* PilY1. This protein, which is exposed on the cell surface, activates virulence features such as pilus biogenesis, twitching motility, secretion of secondary metabolites, and biofilm formation (Bohn et al., 2009; Kuchma et al., 2010; Siryaporn et al., 2014). In *P. aeruginosa* the minor pilins (FimU, PilVWXE) prime type IVa pilus assembly and promote the surface display of the PilY1 adhesin (Giltner et al., 2010; Heiniger et al., 2010; Luo et al., 2015; Nguyen et al., 2015). *L. pneumophila* possesses a comparable *pilY1* pilin gene cluster; an orthologous set of pilin-like proteins controls type IV pilus dynamics and the localization of PilC in *N. gonorrhoeae* (Winther-Larsen et al., 2005).

Interestingly, PilY1 proteins of *L. pneumophila, P. aeruginosa*, and *K. kingae* have a domain that shares homology with the eukaryotic mechano-sensitive vWFa domain and therefore could serve as a sensor for the mechanical cues associated with surface contact. PilY1 of *P. aeruginosa* is up-regulated upon surface contact, which is necessary for surface-activated virulence. Furthermore, deletion of the putatively mechanosensitive vWFa domain places PilY1 in a constitutively active state, inducing virulence in cells, which are not attached to the surface (Siryaporn et al., 2014). Apparently, shear forces exerted on surface-attached *P. aeruginosa* cells shift the surface adhesion PilY1 into an active stretched state and thus inducing virulence toward a broad range of hosts without relying upon chemical recognition of any specific host factor. Accordingly, we hypothesize that *L. pneumophila* PilY1 may exert related functions in pathogenicity.

*L. pneumophila* pathogenesis in lung tissue typically depends on intracellular growth of the pathogen and bacterial dissemination within the tissue. In the present study, we showed that *L. pneumophila* PilY1 is a cell-surface-associated protein which is expressed during the highly virulent stationary growth phase. The presence of PilY1 on the bacterial surface during the transmissive phase of the pathogen seems to be a pre-requisite for a putative mechanosensitive virulence factor or regulator. Since *L. pneumophila* encounters many different surface structures during human infection, we analyzed the effect of PilY1 on the infection of HLTEs. This complex infection model, which comprises a multitude of cell types and extracellular components, including lung epithelial cells, macrophages, and extracellular matrix (ECM), revealed that the *L. pneumophila* Δ*pilY1* mutant has a significant growth defect. In part, this defect seems to be due to the attenuated intracellular growth of the PilY1-negative mutant in phagocytic host cells. This is suggested by previous infections of U937-macrophage-like cells with the PilY1-negative Mini-Tn10 transposon mutant Lpc2666 (Shevchuk et al., 2014). In the present study, we further substantiated this conclusion using infection experiments with site-directed mutants in THP-1 macrophage-like cells and A549 lung epithelial cells. The intracellular growth defects that were observed further confirmed a multifaceted effect of PilY1 on infection processes ranging from adherence and uptake in non-phagocytic to additionally intracellular replication in phagocytic cells. Thus, it is noteworthy that in HLTEs adherence and invasion related processes strongly influence the outcome of the infection of intracellularly replicating *L. pneumophila*.

Studies performed with *P. aeruginosa, Neisseria*, and *K. kingae* revealed a strong influence of PilY1 and PilC on adherence to epithelial cells (Kehl-Fie et al., 2008; Cheng et al., 2013; Porsch et al., 2013). Since intracellular growth of *L. pneumophila* is a result of adherence, invasion, and intracellular replication, we further dissected the functional contributions of PilY1 to these processes. We found that PilY1 of *L. pneumophila* is required for host cell adherence to THP-1 macrophage-like and A549 lung epithelial cells. As adhesion is critical for host cell invasion and both processes are critical for the intracellular life cycle, these effects, including the resulting decrease in growth rate within phagocytic host cells may be linked to each other. This is certainly true for non-phagocytic A549 epithelial cells and in a broader sense for the HLTE infection model, where absence of PilY1 resulted in a drastic decrease in invasive capacity. Complementation of PilY1-negative mutants with single domain deletion variants revealed that the C-terminal PilY1 domain was essential for adherence to THP-1 and A549 cells, whereas the vWFa domain contributes to invasion of non-phagocytic cells. These results resemble observations made in *P. aeruginosa* where the deletion of the calcium binding C-terminal PilC domain abrogated surface-induced virulence, mimicking the loss of the full-length PilY1 protein, whereas PilY1 with a vWFa domain deletion induced virulence in a constitutive manner (Orans et al., 2010; Siryaporn et al., 2014). In analogy, we assume that the vWFa domain of *L. pneumophila* may also have primarily regulatory functions.

For interpretation of the cellular infection data of the PilY1-negative strains, it should be taken into consideration that the intracellular growth defect may be multifactorial and not only related to type IV pili biogenesis. Originally the corresponding Mini-Tn10 transposon mutant Lpc2666 was screened for its failure to inhibit the fusion of LCVs with lysosomes; fluorescence microscopy demonstrated a significantly higher co-localization ratio with lysosomes, an observation that could be confirmed with the targeted *pilY1* knock-out mutant of the current study (Shevchuk et al., 2014, and Supplementary Figure 2). Furthermore, when repeated in THP-1 macrophage-like and A549 lung epithelial cells, these studies confirmed the differential involvement of PilY1 during intracellular replication. In macrophages but not in epithelial cells PilY1 contributed to avoidance of phagolysosomal maturation as measured by LAMP-1 co-localization (Figure 3 and Supplementary Figure 3). The inhibition of the fusion of LCVs with lysosomes is an intracellular process which is temporally and spatially distinct from the initial type-IV pili-mediated attachment to the host cell surface. Thus, while the effects of adherence and invasion can be explained solely by the intimate attachment of the type IV-pili to the host cell surface, the avoidance of lysosomal degradation could also include some kind of interference with host cell type specific signaling or even a type IV-pili-independent regulation of other bacterial virulence factors.

To further dissect the PilY1-mediated virulence mechanisms and to support our hypothesis that PilY1 plays similar roles in *L. pneumophila* and *P. aeruginosa*, we analyzed the surface motility of *L. pneumophila*. Surface motility allows pathogens to migrate over mucosal epithelia and to disperse to other anatomical sites within the host (Kearns, 2010; Taylor and Buckling, 2011; Burrows, 2012). *L. pneumophila* and *P. aeruginosa* can generally exhibit three distinct surface-associated motile behaviors, namely, flagellum-propelled swarming motility, type IV pilus-mediated twitching motility, and surfactant-mediated passive sliding motility (Bohn et al., 2009; Coil and Anné, 2009; Stewart et al., 2009). In *P. aeruginosa*, the minor pilins PilW and PilX, as well as PilY1 participate in c-di-GMP-mediated repression of swarming motility. Moreover, it has been shown that biofilm formation and swarming motility are inversely regulated (Kuchma et al., 2012). In twitching motility of *P. aeruginosa*, PilY1 functions as a calcium-dependent regulator which is essential for type IV pilli biogenesis (Carbonnelle et al., 2006; Heiniger et al., 2010; Orans et al., 2010; Johnson et al., 2011; Cheng et al., 2013; Porsch et al., 2013). Sliding motility of *P. aeruginosa* is observed in the absence of type IV pili and responds to many of the same regulatory proteins and environmental cues as swarming motility (Murray and Kazmierczak, 2008). We demonstrated that *Legionella* PilY1 positively influences twitching motility, but down-regulates sliding motility. The positive effect of PilY1 on twitching motility can be explained by a facilitated type IV pilus assembly. The observed suppression of sliding motility, however, cannot simply be explained by pilus biogenesis. Possible explanations are that PilY1-negative mutants are more leaky for surfactants, or that type IV pili-mediated attachment hampers sliding motility, or that PilY1 down- or up-regulates other effector molecules of *L. pneumophila*.

## Conclusion

In the present study, we characterized PilY1 as a new virulence factor of *L. pneumophila*, a factor which influences intracellular growth and surface motility of the pathogen. We propose that PilY1 functions as an adhesion factor, which not only influences bacterial uptake by host cells, but also the successful establishment of the early LCV by providing close cell-cell contact for effective effector translocation. Whether or not PilY is a master mediator in the hierarchy of virulence regulation, as was proposed for *P. aeruginosa*, remains speculative. The observed effects of PilY1 on adherence, invasion, and twitching motility of *L. pneumophila* can theoretically be explained by their contribution to type IV pili biogenesis. The interference with lysosomal degradation and the suppression of sliding motility have not yet been studied extensively enough to classify them as type IV pili-independent. Similarly, it is an open question if the vWFa domain of PilY1 exerts regulatory functions on the intracellular growth cycle and on tissue dissemination of the pathogen via assembly of type IV pili. Since bacterial cells which are attached to the surface are subjected to larger shear forces than planktonic cells when liquid flows over them, it is appealing to speculate on a mechanosensing function of the vWFa domain. For *P. aeruginosa*, the detection of mechanical cues associated with surface attachment is independent of both the respective surface and the host (Siryaporn et al., 2014; Ellison and Brun, 2015). This enables the pathogen to induce virulence toward a broad range of hosts. Future studies will show if *L. pneumophila* utilizes a similar mechanism to infect a plethora of protozoa species and human cell types.

## Author contributions

JH was involved in all experimental procedures and statistical analysis. CÜ participated in the cloning experiments and contributed to the design of the study. ST and LG performed co-localization and immunofluorescence studies. TG, NG, and MR were responsible for the generation of lung tissue explants (this study is in accordance with the Helsinki declaration and approved by the ethic committee of the Hannover Medical School; No: 2235-2014) and contributed their expertise with the HLTE infection model. OS and MS were involved in the experimental design, data analysis and preparation of the manuscript.

## Funding

The work received financial support from the Deutsche Forschungsgemeinschaft (DFG STE 838/8-1).

### Conflict of interest statement

The authors declare that the research was conducted in the absence of any commercial or financial relationships that could be construed as a potential conflict of interest.
