# Supplementary material for: PilY1 Promotes Legionella pneumophila Infection of Human Lung Tissue Explants and Contributes to Bacterial Adhesion, Host Cell Invasion, and Twitching Motility
Source: Front Cell Infect Microbiol. 2017 Mar 7;7:63. doi: 10.3389/fcimb.2017.00063 (PMC5339237; doi:10.3389/fcimb.2017.00063)
Supplement: Supplementary file 1 [file DataSheet1.pdf]

SUPPLEMENTARY INFORMATION

Material and Methods:

Measuring co-localization of phagolysosomal markers with *Legionella*-containing vacuoles (LCVs) by immunofluorescence microscopy

Mannose-sulfate (M6S) was used as a phagosomal marker in *Dictyostelium discoideum*, whereas LAMP-1 was used in the human cell lines THP-1 and A549 (Newton *et al.,* 2007). Infection and detection of (M6S) on LCVs was performed as described previously (Shevchuk *et al.,* 2014). Briefly, $4 \cdot 10^5$ *Dictyostelium discoideum* cells were seeded onto glass cover slips placed in the wells of a 24-well plate, and were left overnight at 24 °C for attachment. Plate grown bacteria were adjusted to $4 \cdot 10^9$, stained with 5(6)-Carboxytetramethylrhodamine (Sigma-Aldrich) according to the protocol of Fajardo *et al,* 2004, adjusted to $2 \cdot 10^7$ cfu/ml, and 40 µl of these suspensions were added to the cells (MOI 200). In order to synchronize the infection process, bacteria were centrifuged on the cells (233 *g*, 5 min, RT). After 4 h of infection at 25 °C, the cells were washed three times with SorC, and fixed with ice cold methanol for 30 min at 4 °C. Following fixation, the cells were washed three times with SorC buffer, and blocked using 2 % normal human serum in SorC (blocking buffer). For detecting phagolysosomes an antibody recognizing the marker M6S (kindly provided by Markus Maniak) was applied to the cells for 1 h at RT in a humidified chamber. Unbound antibodies were removed by three washes of blocking buffer, and the cells were covered with SorC containing 1 % (w/v) BSA and Alexa Fluor® 488 conjugated polyclonal α-mouse-antibodies (Abcam). After 1 h at RT, excess antibodies were removed by washing three times with blocking buffer, and the DNA was stained with DAPI (Sigma-Aldrich) diluted 1:1000 in PBS. Infection of the human THP-1 and A549 cells with rhodamine labeled bacteria was performed as described in material and methods. Detection of LAMP-1 was performed as described above with the exception that the cells were fixed with 4 % PFA in PBS, and a mouse α-LAMP-1 antibody (diluted 1:200) was used. The preparations were covered using fluorescence mounting medium from Dako (S3023). Samples were analyzed and images were taken using a Leica SP8 confocal laser scanning microscope. Co-localization of rhodamine-labeled bacteria with M6S was measured using ImageJ (Rasband W.S.). Data visualization and statistical analysis was performed sing Graphpad Prism 5.0.

References:

Fajardo, M., Schleicher, M., Noegel, A., Bozzaro, S., Killinger, S., Heuner, K., Hacker, J., Steinert, M. (2004). Calnexin, calreticulin and cytoskeleton-associated proteins modulate uptake and growth of *Legionella pneumophila* in *Dictyostelium discoideum*. *Microbiology*. 150, 2825-2835.

Rasband, W.S., ImageJ, U. S. National Institutes of Health, Bethesda, Maryland, USA, http://imagej.nih.gov/ij/, 1997-2016.

Shevchuk, O., Pägelow, D., Rasch, J., Döhrmann, S., Günther, G., Hoppe, J., Ünal, C.M., Bronietzki, M., Gutierrez, M.G., Steinert, M. (2014). Polyketide synthase (PKS) reduces fusion of *Legionella pneumophila*-containing vacuoles with lysosomes and contributes to bacterial competitiveness during infection. *Int J Med Microbiol*. 304, 1169-1181.

Swanson, M.S., Isberg, R.R. (1996) Identification of *Legionella pneumophila* mutants that have aberrant intracellular fates. *Infect Immun*. 64(7), 2585-2594.

Supp. Fig. 1

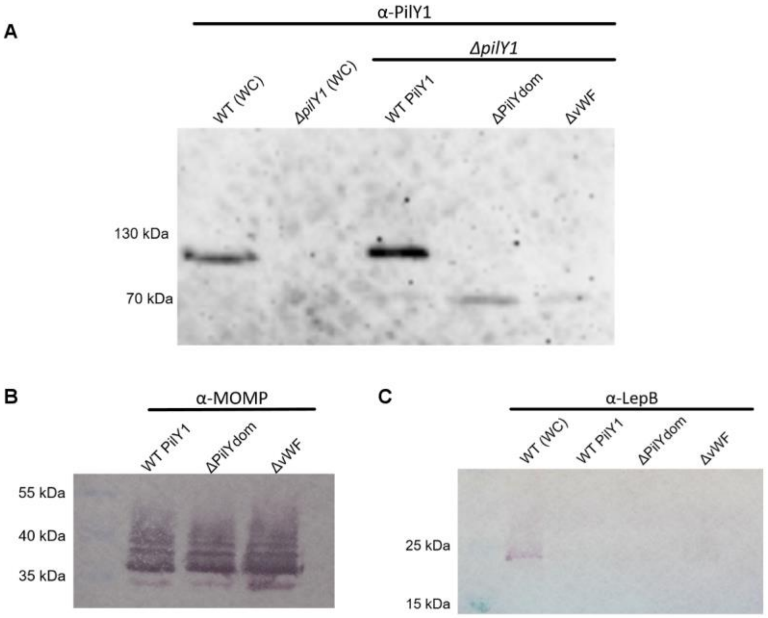

Supp. Fig. 1: PilY1 and its variants localize at the surface of *L. pneumophila*. (A) Triton X-100 insoluble outer membane fractions of the complementation strains were checked for the presence of wild type PilY1 and its domain deletion variants by western blot. As controls whole cell lysates (WC) of *L. pneumophila* and its *ΔpilY1* mutant were used. Wild type PilY1 as well as the variant lacking the PilY-domain (ΔPilYdom) were detected in these fractions at the expected sizes of 120 and 60 kDa. The variant lacking the vWFa-domain could not be detected at its expected size of 100 kDa due to the loss of the centrally located peptide epitope recognized by the antibody 927 1-2. during cloning (B) The fractions were confirmed as being OM with a western blot, where the major outer mambrane protein (MOMP) was detected. (C) All fractions were shown to be free of inner membrane components with a western blot, in which the presence of the signal peptidase LepB was used as a control. Whole cell lysate of wild type *L. pneumophila* served as a positive control.

Supp. Fig. 2

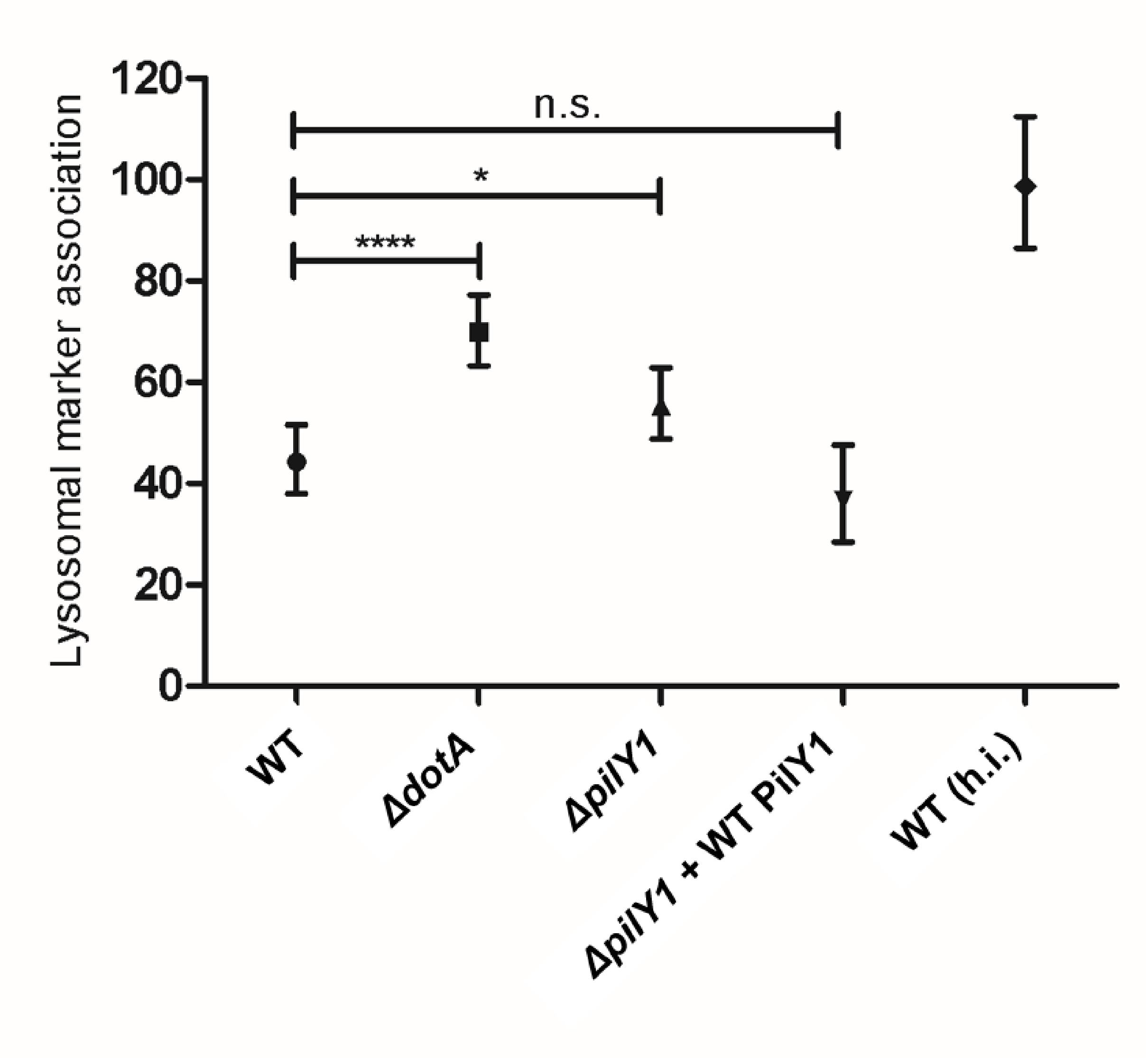

Supp. Fig. 2: PilY1 contributes to the avoidance of phagolysosome formation. Co-localization of rhodamine labeled *L. pneumophila* Corby strains with the phagolysosomal marker mannose-6-sulfate (M6S) was measured by immunofluorescence staining using an Alexa Fluor® 488 labeled secondary antibody. The integrated density of green fluorescence at the site of the bacteria was determined using the image analysis tool ImageJ and divided by the respective area, resulting in lysosomal marker association ratios. The experiments were performed three times and for each strain a minimum of 100 intracellular bacteria were analyzed. Shown are the geometrical means with the 95 % confidence intervals. Each strain was compared to the wild type strain using a unpaired, two sided Mann-Whitney test (*$p\leq0{,}05$, ****$p\leq0{,}0001$, n.s., no significance).

Supp. Fig. 3

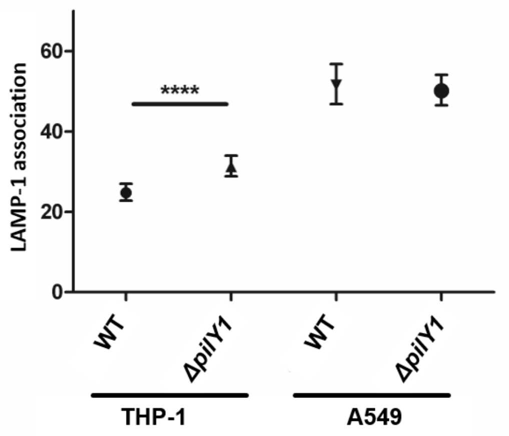

Supp. Fig. 3: PilY1 contributes to the avoidance of phagolysosome formation in macrophages but not in epithelial cells. Co-localization of rhodamine labeled *L. pneumophila* Corby strains with the phagolysosomal marker LAMP-1 was measured by immunofluorescence staining using an Alexa Fluor® 488 labeled secondary antibody. The integrated density of green fluorescence at the site of the bacteria was determined using the image analysis tool ImageJ and divided by the respective area, resulting in lysosomal marker association ratios. The experiments were performed three times and for each strain a minimum of 100 intracellular bacteria were analyzed. In the human macrophage cells THP-1, PilY1-deficient bacteria co-localized significantly more with LAMP-1 indicating that they are not able to inhibit fusion of phagosomes with lysosomes, a hallmark of the intracellular life cycle. In the epithelial cell line A549, on the other hand, no significant difference was observed between wild type and its isogenic *ΔpilY1* mutant. Shown are the geometrical means with the 95 % confidence intervals. Significance was assessed using an unpaired, two sided Mann-Whitney test (*****p≤0,0001).

Supp. Fig. 4

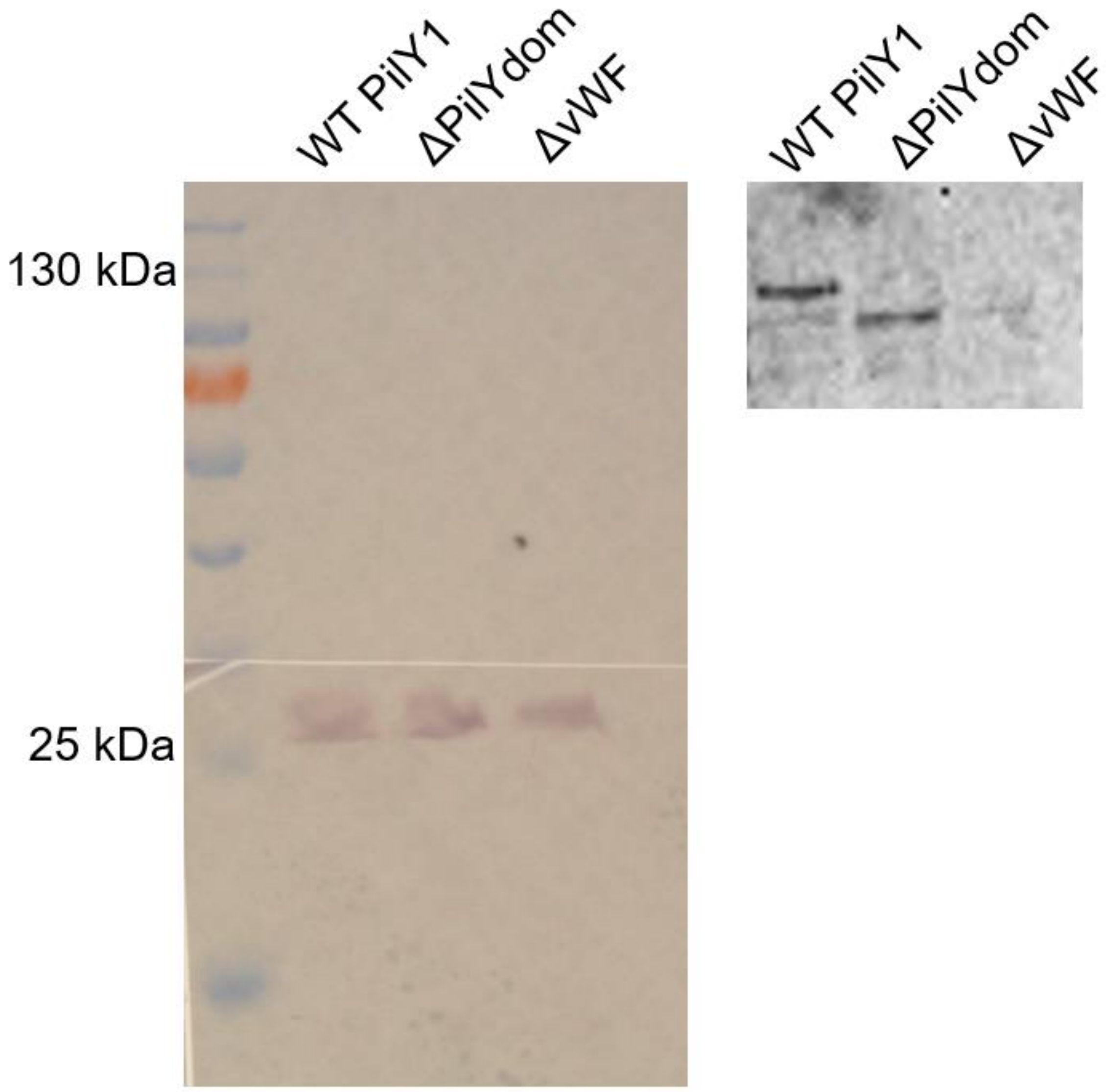

Supp. Fig. 4: PilY1 and its variants localize at the surface of *L. pneumophila*. Localization in the OM was further confirmed using the outer membrane protein Mip as a marker and loading control. Equal amounts of protein were separated by SDS-PAGE using 12 % acrylamide. After blotting the membrane was cut in two in order to detect Mip using an α-mouse antibody coupled to alkaline phosphatase. PilY1 and its variants were detected using an α-rabbit antibody coupled to horseradish peroxidase.
